# GRP78 Inhibitor YUM70 Suppresses SARS-CoV-2 Viral Entry, Spike Protein Production and Ameliorates Lung Damage

**DOI:** 10.3390/v15051118

**Published:** 2023-05-06

**Authors:** Dat P. Ha, Woo-Jin Shin, Juan Carlos Hernandez, Nouri Neamati, Louis Dubeau, Keigo Machida, Amy S. Lee

**Affiliations:** 1 Department of Biochemistry and Molecular Medicine, Keck School of Medicine, University of Southern California, Los Angeles, CA 90033, USA; datha@usc.edu; 2 Norris Comprehensive Cancer Center, Keck School of Medicine, University of Southern California, Los Angeles, CA 90033, USA; 3 Florida Research and Innovation Center, Cleveland Clinic, Port St. Lucie, FL 34987, USA; 4 Department of Molecular Microbiology and Immunology, Keck School of Medicine, University of Southern California, Los Angeles, CA 90033, USA; 5 Department of Medicinal Chemistry, College of Pharmacy and Rogel Cancer Center, University of Michigan, Ann Arbor, MI 48109, USA; 6 Department of Pathology, Keck School of Medicine, University of Southern California, Los Angeles, CA 90033, USA

**Keywords:** GRP78, BiP, ER stress, COVID-19, SARS-CoV-2, variants, YUM70, small molecule inhibitor

## Abstract

The severe acute respiratory syndrome coronavirus 2 (SARS-CoV-2), the causative agent of the COVID-19 pandemic, has given rise to many new variants with increased transmissibility and the ability to evade vaccine protection. The 78-kDa glucose-regulated protein (GRP78) is a major endoplasmic reticulum (ER) chaperone that has been recently implicated as an essential host factor for SARS-CoV-2 entry and infection. In this study, we investigated the efficacy of YUM70, a small molecule inhibitor of GRP78, to block SARS-CoV-2 viral entry and infection in vitro and in vivo. Using human lung epithelial cells and pseudoviral particles carrying spike proteins from different SARS-CoV-2 variants, we found that YUM70 was equally effective at blocking viral entry mediated by original and variant spike proteins. Furthermore, YUM70 reduced SARS-CoV-2 infection without impacting cell viability in vitro and suppressed viral protein production following SARS-CoV-2 infection. Additionally, YUM70 rescued the cell viability of multi-cellular human lung and liver 3D organoids transfected with a SARS-CoV-2 replicon. Importantly, YUM70 treatment ameliorated lung damage in transgenic mice infected with SARS-CoV-2, which correlated with reduced weight loss and longer survival. Thus, GRP78 inhibition may be a promising approach to augment existing therapies to block SARS-CoV-2, its variants, and other viruses that utilize GRP78 for entry and infection.

## 1. Introduction

SARS-CoV-2, the virus that causes the COVID-19 pandemic, remains a major global public health concern. While the rapid development of vaccines has significantly reduced severity and mortality, new variants of SAR-CoV-2 continue to appear. For example, the Omicron variant, which emerged in November 2021, has many lineages which have spread in the United States and globally, posing challenges to the existing vaccine strategy and evading treatment [1]. As it may not be possible to eliminate SARS-CoV-2, an alternative to pursuing the constantly evolving virus is to target the crucial host factors required for viral entry and infection that are more stable and less prone to mutation, rather than pursuing the constantly evolving virus.

Viruses obligate intracellular parasites, which depend entirely on their host’s cellular machinery to manufacture the genome and proteins required for virion production, assembly, and budding [2,3,4]. Additionally, many viruses, including SARS-CoV-2, are enveloped by a lipid bilayer containing viral surface glycoproteins that bind to host cell receptors and facilitate viral entry [3,4]. Since these viral envelope proteins are membrane-embedded, they are synthesized and processed in the endoplasmic reticulum (ER), which is a major site for the synthesis, folding, and maturation of membrane and-secreted proteins [2,4]. The ER houses a diverse repertoire of molecular chaperones to aid in the folding process and/or direct the degradation of misfolded proteins [5]. When the folding capacity of the ER is overwhelmed due to increased protein synthesis or excessive amounts of misfolded proteins, the cell undergoes ER-stress which activates the unfolded protein response (UPR): a complex network of signaling pathways that aim to restore ER homeostasis [6,7,8].

The 78-kDa glucose-regulated protein (GRP78), also known as BiP or HSPA5, is a major molecular chaperone residing in the ER with critical roles in protein folding, impacting health and diseases, and acts as the master regulator of the UPR [6,7,9]. Knockdown or the inhibition of GRP78 activity triggers ER stress and activates the UPR, including transient global translational arrest mediated by the PERK/eIF2α pathway [6,7,8]. Although GRP78 has traditionally been regarded as an ER luminal protein, there is growing evidence that GRP78 can also be detected on the cell surface under stressed conditions and assumes novel functions as a receptor that controls signaling, proliferation, invasion, apoptosis, inflammation, immunity, and viral infection [7,10,11,12,13,14,15]. Since ER stress is a common aspect of cancer and viral infection due to the increased demand for newly synthesized proteins to support cell proliferation or virus production, GRP78 has emerged as a key target to combat these diseases [4,7,9,16,17,18]. Recently, we and others have demonstrated that GRP78 expression is increased in SARS-CoV-2-infected cells and patients and that targeting GRP78 could disrupt multiple stages of the viral life cycle, including entry, production, and subsequent cellular infection [15,19,20,21,22,23,24]. In Carlos et al., we showed that GRP78 can form a complex with the SARS-CoV-2 spike protein and ACE2 receptor and that GRP78 knockdown reduces cell surface ACE2 expression [15]. Importantly, the inhibition of cell surface GRP78 by the monoclonal antibody mAb159 could block SARS-CoV-2 entry in a pseudo-virus entry assay as well as in live viral infections in vitro [15]. In Shin et al., we established that SARS-CoV-2 infection led to an increase in both GRP78 mRNA and protein levels in a temporal manner [24]. Additionally, as proof of principle, GRP78 depletion by siRNA knockdown followed by infection with live SARS-CoV-2 in two different cell lines remarkably diminished virus production in the plaque formation assay [24]. Importantly, treatment with the GRP78 inhibitor HA15 also resulted in the dramatic suppression of viral infection in both in vitro and in vivo model systems without affecting cell viability [24]. Taken together, these and other studies demonstrated that targeting GRP78 might block SARS-CoV-2 infection. The question remains whether targeting GRP78 is effective against the variants of SARS-CoV-2 and whether other GRP78 inhibitors can also confer beneficial effects on the infected host.

The hydroxyquinoline analog YUM70 was discovered as a selective inhibitor of GRP78 that could induce ER stress and block pancreatic cancer cell growth in vitro and in vivo with no detectable toxicity to normal tissues [25]. YUM70 was identified in a phenotypic screen for cytotoxicity from 40,000 in-house drug-like compounds and was selected for its superior solubility, microsomal stability, and pharmacokinetic properties [25]. Further biochemical analyses showed that YUM70 bound to recombinant and cellular GRP78 and was selective for GRP78 over other ER proteins, including GSTO1, PDI, and the closely related cytosolic chaperone HSP70 [25]. In a recent study, we also found that the inhibition of GRP78 by YUM70 could suppress oncogenic KRAS protein expression and reduce the viability of lung, colon, and pancreatic cancer cells bearing various KRAS mutations [26]. Mechanistically, YUM70 directly bound to GRP78 and inhibited its enzymatic activity, leading to ER stress and UPR-mediated apoptosis via the PERK/eIF2a/ATF4/CHOP pathway [25]. In the current study, we examined whether YUM70 was effective at blocking SARS-CoV-2 and variant viral particle entry and the expression of ectopic and endogenous spike protein production. We further investigate the effect of YUM70 on cell viability in 3D lung and liver organoids and lungs following SARS-CoV-2 infection in mice.

## 2. Materials and Methods

### 2.1. Immunoblot Analysis

Cells were lysed in cold RIPA buffer (50 mM Tris-HCl, 150 mM NaCl, 1% NP-40, 0.5% sodium deoxycholate, and 0.1% SDS) supplemented with a Protease and Phosphatase inhibitor cocktail (ThermoFisher, Waltham, MA, USA, Cat#78441). The cell lysates were then incubated on ice for 30 min followed by centrifugation at 13,000 RPM at 4 °C for 15 min. The supernatants containing soluble proteins were saved as the clarified cell lysates for subsequent biochemical analysis. Proteins were electrophoresed in 8% SDS-PAGE gels and transferred to a nitrocellulose membrane at 30 V overnight at 4 °C. The membrane was blocked with 5% non-fat dry milk in Tris-buffered saline with 0.2% Tween-20 (TBST) for 1 h, followed by primary antibody incubation in 5% BSA TBST overnight. The next day, the membrane was washed three times with TBST and incubated with an HRP-conjugated secondary antibody for 2 h. The following primary antibodies were used in this study: mouse anti-SARS-CoV-2 spike protein (S1-NTD) (1:1000, Cell Signaling, Danvers, MA, USA, #42172), rabbit anti-SARS-CoV-2 Nucleocapsid protein (E9L7H) (1:1000, Cell Signaling, #86326), mouse anti-β-actin (1:1000, Santa Cruz Biotechnology, Inc., Dallas, TX, USA, sc-8432). Secondary antibody: mouse IgG1 binding protein conjugated to HRP (1:1000, Santa Cruz Biotechnology, Inc., sc-525408), mouse anti-rabbit IgG conjugated to HRP (1:1000, Santa Cruz Biotechnology, Inc., sc-2357). HRP signal was detected by SuperSignal West Pico Chemiluminescence substrate (ThermoFisher, Cat# 34080) and protein bands were visualized by ChemiDoc XRS+ imager (Bio-Rad Laboratories, Hercules, CA, USA) and quantified by Image Lab software version 4.0.1 build 6 (Bio-Rad Laboratories).

### 2.2. Cell Culture and Drug Treatment

The African green monkey kidney epithelial cell line Vero E6 ACE2 was cultured in Dulbecco’s modified Eagle medium (DMEM) containing 10% fetal bovine serum (FBS; Gemini Bio, West Sacramento, CA, USA), 1% penicillin/streptomycin (pen/strep) (Corning Inc., Glendale, AZ, USA), and 1 μg/mL puromycin. The human embryonic kidney 293T (HEK 293T) cell line was cultured in an DMEM containing 10% FBS and 1% pen/strep. The human non-small cell lung adenocarcinoma cell line H1299 was cultured in a RPMI 1640 medium containing 10% FBS and 1% pen/strep. All cell lines were maintained at 37 °C in a humidified atmosphere of 5% CO_2_ and 95% air. YUM70 was dissolved in DMSO to prepare a 10 mM stock solution, and aliquotes were made in culture media. Vero E6 ACE2 cells were treated with YUM70 at indicated concentrations, and 1% DMSO was used as a control. The Vero E6 ACE2 cell line was a generous gift from Younho Choi at USC. The H1299 cell line was obtained from ATCC.

### 2.3. Expression Vector and Transfection

The expression vector for the SARS-CoV-2 spike protein was a generous gift from Dr. Stefan Pohlmann (Leibniz Institute for Primate Research, Gottinggen, Germany) [27]. The expression vector for the Southern California variant strain S protein was generated by subcloning from gBlock, and the Omicron variant strain S protein was subcloned from Addgene plasmids (#183700) with a restriction enzyme digestion method. Transfections of plasmids were performed with Lipofectamine 3000 Transfection Reagent (Thermo Fisher Scientific, Cat#L3000015) following the manufacturer’s instructions.

### 2.4. Generation of VSV Pseudotyped Viral Particles and Transduction Experiments

The procedure for VSV pseudo-particle production followed a published protocol [28]. Briefly, HEK293T cells, ectopically expressing the original SARS-CoV-2-spike or other spike variants, were inoculated with a replication-deficient VSV vector, VSVΔG-fLuc (kindly provided by Jae Jung, Department of Molecular Microbiology and Immunology, USC, CA, USA) that expressed firefly luciferase instead of VSV-G. After 1 h of incubation at 37 °C, the medium was removed, and cells were washed twice with PBS before fresh complete media was added. Twenty-four hours post-inoculation, a medium containing pseudotyped particles was harvested and centrifugated. The supernatant clarified from cellular debris was used in transduction experiments. For transduction, H1299 cells grown to 70% confluency in 96-well plates were inoculated with indicated pseudo particles. To inhibit GRP78 activity, cells were treated with the indicated dose of YUM70 2 h before transduction. DMSO was used as vehicle control. At 16 h post-transduction, the firefly luciferase activity was determined using ONE-Glo™ Luciferase Assay System (Promega, Medison, WI, USA) and a FLUOstar Omega microplate reader (BMG Labtech, Durham, NC, USA). The experiment was repeated 3 times.

### 2.5. WST-1 Cell Viability Assay

Vero E6 ACE2 cells were seeded at a density of 10,000 cells per well in a 96-well plate with DMEM supplemented with 10% FBS and 1% pen/strep. The cells were allowed to be attached overnight, and the next day, the medium was removed and replaced with serum-free DMEM containing 1% pen/strep and no FBS. The cells were then treated with DMSO, containing increasing concentrations of YUM70 (0 μM to 10 μM). Cell viability was measured at 24, 48, and 72 h post-drug treatment using the (4-[3-(4-Iodophenyl)-2-(4-nitro-phenyl)-2H-5-tetrazolio]-1,3-benzene sulfonate) WST-1 cell proliferation assay kit (Takara Bio USA, Inc., San Jose, CA, USA) according to the manufacturer’s recommendation. Colorimetric quantitation was achieved using a Model 680 Microplate Reader (Bio-Rad Laboratories, Hercules, CA, USA) at a wavelength of 450 nm and subtracted by a reference wavelength of 650 nm. The background absorbance of blank media (DMEM 1% pen/strep) was also measured and subtracted from the sample reading. The experiment was repeated 4 times.

### 2.6. Virus Propagation

The following reagent was deposited by the Centers for Disease Control and Prevention and obtained through BEI Resources, NIAID, NIH: SARS-Related Coronavirus 2, Isolate USA-WA1/2020, NR-52281. The virus was propagated in Vero E6 ACE2 in a DMEM media supplemented with 10% FBS, 1% penicillin-streptomycin (Gibco) and 0.5 µg/mL TPCK-treated trypsin (Worthington Biochemical). When 90% CPE was confirmed, the supernatant was collected and passed through a 0.45-micron pore-size polyethersulfone (PES) filter and aliquoted and stored at −80 °C until further use. The virus titer was determined by a plaque assay.

### 2.7. Plaque Reduction Assay

The anti-viral activity of YUM70 was evaluated by the plaque reduction assay as described previously [29]. Confluent monolayers of Vero E6 ACE2 cells in 6-well plates were washed once with DMEM and infected with approximately 100 plaque-forming units (PFUs) of SARS-CoV-2 in each well. Virus-free DMEM was used for mock infections. The plates were incubated at 37 °C for 45 min for viral adsorption. The virus inoculum was then removed and replaced by an overlay medium (DMEM containing 1% low-melting agarose without serum) containing 2-fold serial dilutions of YUM70 and were placed in a 37 °C CO_2_ incubator for 72 h until the plaques could be visualized under a light. The cells were fixed in a 4% formaldehyde solution for at least 30 min, and the overlaid agarose was removed. The cells were stained with a 0.2% (*w/v*) crystal violet solution. The plaques were counted by visual examination, and their sizes were measured by scale loupe. The experiment was repeated 3 times.

### 2.8. Virus Infection, Drug Treatment and Cell Harvest

Vero E6 ACE2 cells were seeded in 6-well plates and allowed to attach overnight. The cells were washed once with fresh DMEM and infected with 0.5 or 3 MOI of SARS-CoV-2 in each well. The plates were incubated on a rocker at 37 °C for 45 min for virus adsorption. The virus inoculum was then removed and replaced by fresh DMEM media. The cells were then treated with the indicated doses of YUM70 and placed in a 37 °C CO_2_ incubator. The cell pellets were collected at indicated time points and stored at −80 °C. The cell pellet samples were lysed, and cell lysates were subjected to immunoblot analysis as described above. The experiment was repeated 3 times.

### 2.9. Animals and In Vivo Procedures

K18-hACE2 transgenic mice were purchased from Jackson Laboratory (cat. no. 034860) and bred and maintained in specific-pathogen-free facilities at USC. Genotyping was performed with primers from Jackson Laboratory. Hemizygous, 8–10 weeks old male and female mice were used. Mice were housed under a 12:12 light: dark cycle in facilities accredited by the Association for Assessment and Accreditation of Laboratory Animal Care International. All animal care and experiments were performed according to the NIH guidelines for the care and use of laboratory animals. All animal studies were approved by the Institutional Animal Care and Use Committee of USC.

For in vivo SARS-CoV-2 infection, K18-hACE2 transgenic mice were first transferred into the ABSL3 facility of USC. All animal procedures (including infection, injection, weighing and euthanasia) were performed after anesthesia by isoflurane. Mice were intranasally infected with 10^4^ pfu of SARS-CoV-2 virus (USA-WA1/2020) in 30 μL serum-free DMEM. YUM70 was first dissolved in a diluent (10% DMSO, 60% propylene glycol, and 30% saline *v/v*) and diluted in PBS for intraperitoneal injection. The same volume of the vehicle buffer (PBS diluted from 10% DMSO, 60% propylene glycol, and 30% saline *v*/*v*) was used in the control groups. YUM70 was intraperitoneally injected daily (30 mg/kg body weight in PBS diluted from 10% DMSO, 60% propylene glycol, and 30% saline *v/v* stock solution) one day before infection and five days per week post-infection. On days 3 and 5, a subset of mice was euthanized by carbon dioxide inhalation, followed by cervical dislocation, and tissue samples were collected for histopathological analysis. The left lung of each mouse was fixed in buffered formalin for sectioning and H&E staining. The vehicle control and YUM70 treatment groups contained 5 mice each.

### 2.10. Measurement of Magnitude of Pulmonary Damage

One lung from each mouse was fixed in buffered formalin and embedded in paraffin. A total of 4 micron-thick histological sections were obtained from each paraffin block. The sections were stained with hematoxylin and eosin, digitized using a Philips UltraFast scanner, and visualized using the IntelliSite Image Management System 3.2. A grid of 0.5 cm^2^ tiles was placed over each digitized slide. The proportion of damaged alveoli in each lung was estimated by dividing the number of tiles overlying the areas showing inflammation and associated with alveolar wall thickening or collapse by the total number of tiles overlying any alveoli. The standard error was calculated by comparing data from 5 different sections of each lung, each separated by 100 microns of lung tissue.

### 2.11. Statistical Analysis

All pair-wise comparisons were made using the two-tailed unpaired Student’s *t*-test in Microsoft Excel. Data were presented as the mean ± Standard Deviation (S.D.). A *p*-value of ≤0.05 is signified by *, *p*-value of ≤0.01 by **, *p*-value of ≤0.001 by ***.

## 3. Results

### 3.1. GRP78 Inhibitor YUM70 Blocks Pseudovirus Entry Mediated by Different SARS-CoV-2 Variants Spike Proteins

**Figure 1:**
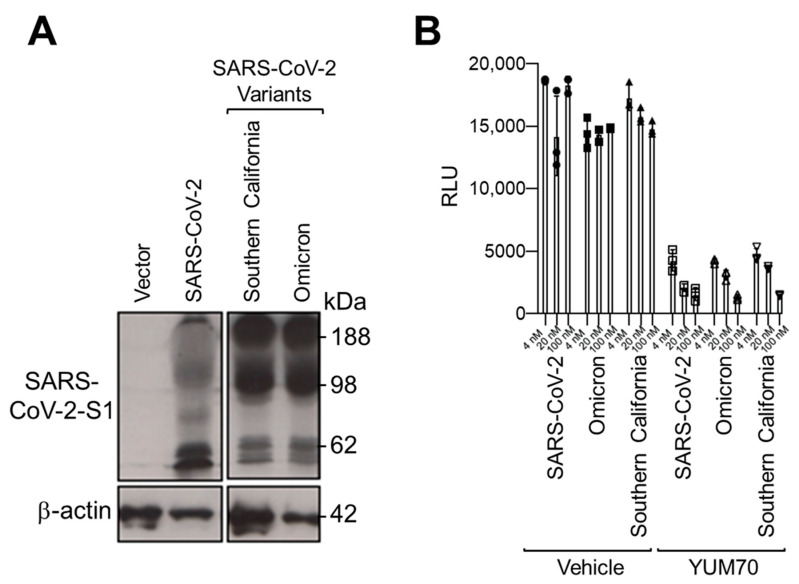
GRP78 inhibitor YUM70 blocks pseudovirus entry mediated by different SARS-CoV-2 variant spike proteins. (**A**) HEK293T cells were transfected with empty vectors or vectors expressing the spike proteins from different SARS-CoV-2 variants for 48 h. Whole-cell lysates were analyzed by Western blot for spike protein level with β-actin serving as the loading control. (**B**) H1299 cells were pre-treated with DMSO or indicated a concentration of YUM70 for 2 h before inoculation with pseudovirus harboring the original or variant SARS-CoV-2 spike protein. At 16 h post-infection, relative luciferase activities were measured in the infected cells and graphed. Data are presented as means ± S.D. (*n* = 3).

To test whether the inhibition of GRP78 by a small molecule can block viral entry mediated by the spike proteins from emerging variants of SARS-CoV-2, we utilized the human lung epithelial cell line H1299 and pseudo viral particles bearing different SARS-CoV-2 spike proteins and a luciferase reporter gene as a viral entry model system. First, HEK293T cells were transfected with empty vectors or vectors expressing the spike proteins from different SARS-CoV-2 variants, and whole-cell lysates were analyzed by Western blot. We observed a robust expression of different spike variants (Figure 1A). Then, pseudo viral particles bearing the original, Omicron or Southern California variants of the SARS-CoV-2 spike protein were produced in HEK29T cells. H1299 cells were transduced with these pseudo particles and treated with vehicle control or increasing doses of YUM70 for 24 h. The luciferase activity assay revealed that YUM70 dramatically reduced the entry of pseudo particles facilitated by the original as well as variant SARS-CoV-2 spike proteins (Figure 1B). These results indicated that, in pseudo-virus entry assays, the inhibition of GRP78 activity by YUM70 was equally effective at blocking the viral entry of the original as well as the SARS-CoV-2 variants that were tested.

### 3.2. YUM70 Blocks SARS-CoV-2 Infection In Vitro without Affecting Cell Viability

**Figure 2:**
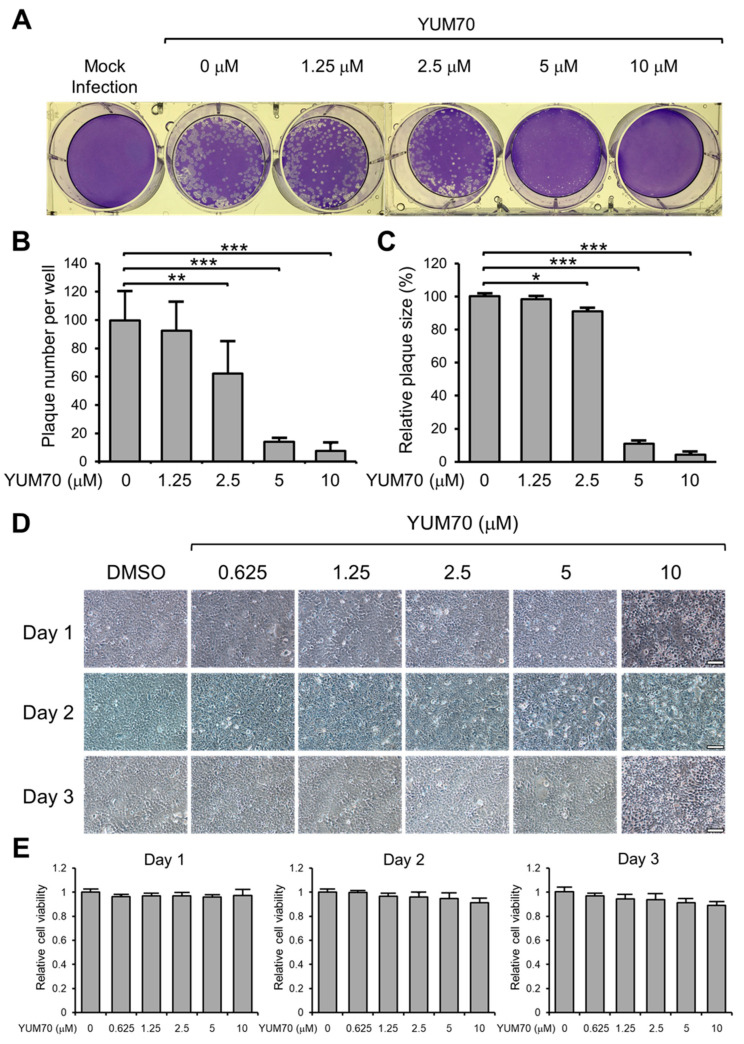
YUM70 suppresses SARS-CoV-2 infection in vitro without affecting cell viability. (**A**) Confluent monolayers of Vero E6 ACE2 cells in 6-well plates were either mock-infected or infected with SARS-CoV-2 virus and treated with DMSO or increasing concentrations of YUM70 for 72 h. The cells were then fixed with 4% formaldehyde and stained with 0.2% crystal violet. The images are representative of three repeats. (**B**) The numbers of plaques in each well from (**A**) were counted and plotted in the graph (*n* = 3). (**C**) The relative plaque size in each well from (**A**) was measured and plotted in the graph (*n* = 7). (**D**) Confluent monolayers of Vero E6 ACE2 cells were treated with DMSO or increasing concentrations of YUM70 for the indicated times. Brightfield microscopy images of the cells were taken. The scale bar represents 100 microns. (**E**) Same as in (**D**) but cell viability was measured by WST-1 assay at 1, 2, and 3 days after drug treatment (*n* = 4). Data are presented as means ± S.D. * *p* ≤ 0.05, ** *p* ≤ 0.01, *** *p* ≤ 0.001 (Student’s *t*-test).

Next, to investigate the inhibitory effect of YUM70 on live SARS-CoV-2 virus infection, we performed the plaque reduction assay in Vero E6 ACE2 cells. The cells were cultured in 6-well plates, infected with a live SARS-CoV-2 virus, and treated with varying concentrations of YUM70 ranging from 0 to 10 μM for 72 h (Figure 2A). This dosage range of YUM70 was chosen based on our previous study showing that the IC50 of YUM70 in human pancreatic cancer cell lines ranged from 2.5 to 10 μM, whereas the IC50 for normal human pancreatic ductal cells was greater than 30 μM [25]. The resulting plaques were quantified, and their sizes were analyzed to determine the degree of infection (Figure 2B,C). Our results indicate that YUM70 efficiently inhibited both the number and size of SARS-CoV-2 plaques formed, with a significant impact observed at 2.5 μM and a dramatic reduction at 5 μM, alongside a nearly complete inhibition at 10 μM (Figure 2A–C).

To rule out the possibility that the observed plaque reduction was due to the decreased cell viability caused by YUM70, cell viability was assessed using brightfield microscopy and a WST-1 assay (Figure 2D,E). The results showed that YUM70 treatment up to 10 μM for 72 h had no significant impact on Vero E6 ACE2 cell viability, suggesting that the plaque reduction effect was not an artifact of the experimental system but was a direct result of YUM70’s ability to inhibit SARS-CoV-2 infection in vitro.

### 3.3. YUM70 Reduces Both Spike and N Protein Levels Following SARS-CoV-2 Infection

**Figure 3:**
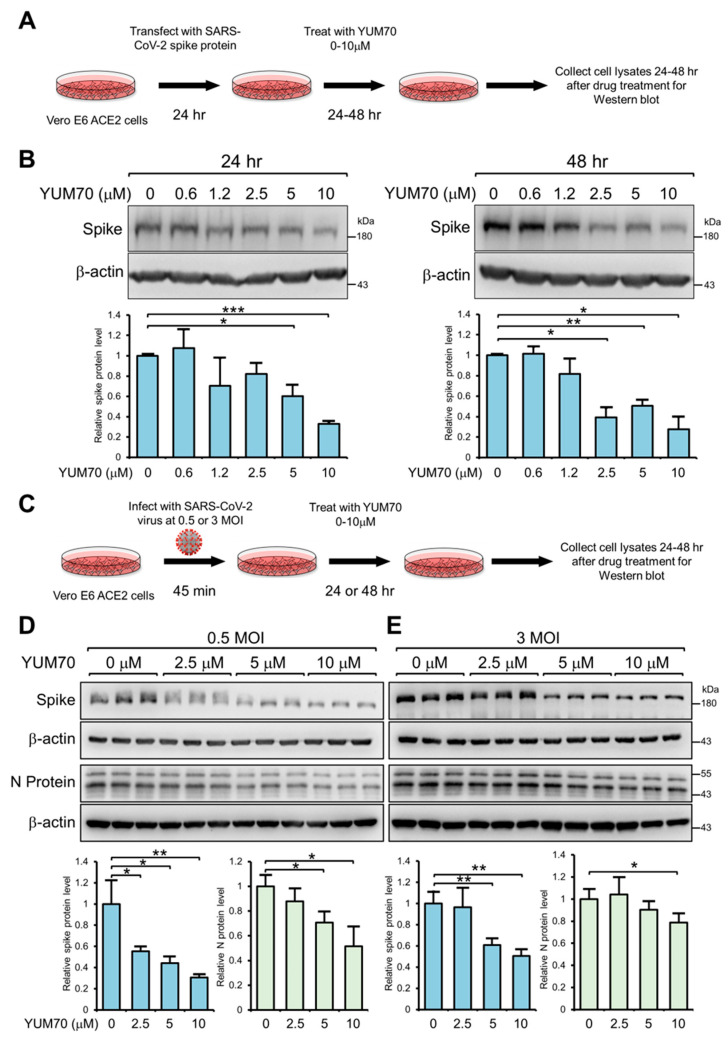
YUM70 reduces spike protein production. (**A**) Schematic diagram of the experimental design for the experiment below. (**B**) Vero E6 ACE2 cells were transfected with a vector expressing the SARS-CoV-2 spike protein for 24 h, followed by treatment of YUM70 at the indicated concentrations for 24 or 48 h. Whole-cell lysates were analyzed by Western blot for spike protein level with β-actin serving as loading control. Quantitation of the relative protein levels of Spike normalized against the β-actin is shown in the graphs below (*n* = 2). (**C**) Schematic diagram of the experimental design for the experiment below. (**D**) Vero E6 ACE2 cells were infected with the SARS-CoV-2 virus at 0.5 MOI, followed by treatment of YUM70 at the indicated concentrations for 48 h. Whole-cell lysates were analyzed by Western blot for spike and N protein level with β-actin serving as loading control. Quantitation of the relative protein levels of spike and N protein normalized against the β-actin is shown in the graphs below (*n* = 3). (**E**) Same as in (**D**), except cells were infected with 3 MOI of SARS-CoV-2 and incubated with YUM70 for 24 h. Data are presented as means ± S.D. * *p* ≤ 0.05, ** *p* ≤ 0.01, *** *p* ≤ 0.001 (Student’s *t*-test).

To elucidate additional mechanisms beyond the viral particle entry that contributed to the ability of YUM70 to inhibit live SARS-CoV-2 infection in vitro, we examined the impact of YUM70 on the production of spike proteins in infected cells. First, we transfected Vero E6 ACE2 cells with an expression vector to overexpress the exogenous spike protein. Following a 24 h transfection period, we treated the cells with increasing concentrations of YUM70 ranging from 0 to 10 μM for 24 or 48 h (Figure 3A). The cells were then harvested, and the levels of the spike protein were assessed using Western blot analysis. The results demonstrated a dose-dependent reduction in the level of the spike protein following treatment with YUM70 at both 24 and 48 h time points (Figure 3B). As expected, the inhibitory effect of YUM70 was more pronounced at a longer time of treatment, such that at 48 h, a 2.5 μM dose of YUM70 was sufficient to significantly reduce the spike protein level (Figure 3B).

Next, we examined the effect of YUM70 on the endogenous production of the spike protein in SARS-CoV-2-infected cells. To achieve this, Vero E6 ACE2 cells were infected with a live SARS-CoV-2 virus at either MOI of 0.5 or 3 for 45 min and subsequently treated with increasing doses of YUM70 ranging from 0 to 10 μM for 24 h for 3 MOI and 48 h for 0.5 MOI (Figure 3C). Following treatment, the cells were collected, and the level of the spike protein was assessed using Western blot analysis. The results showed that YUM70 significantly reduced the level of the endogenous protein produced by live SARS-CoV-2 virus infection in Vero E6 ACE2 cells at an MOI of both 0.5 and 3, with a stronger effect observed at 0.5 MOI (Figure 3D,E). YUM70 effectively reduced the level of endogenously expressed spike protein in a dose-dependent manner at an MOI of 0.5, with statistical significance observed at a 2.5 μM dose of YUM70, while a reduction in the endogenous spike protein was only observed at a dose of 5 μM YUM70 with an MOI of 3 (Figure 3D,E). Since the inhibition of GRP78 by YUM70 activated the UPR, leading to transient global translational arrest beyond the ER, we also examined the effect of YUM70 on the level of viral Nucleocapsid (N) protein, which was synthesized in the cytosol. Similar to spike protein, the N protein level was also reduced by YUM70 treatment in a dosage-dependent manner, albeit with a lesser magnitude than the spike protein (Figure 3D,E). Overall, these findings suggest that YUM70 could effectively suppress the levels of both spike and N protein following SARS-CoV-2 infection.

### 3.4. YUM70 Rescues Cell Viability in Multi-Cellular Human Lung and Liver 3D Organoids Transfected with SARS-CoV-2 Replicon

**Figure 4:**
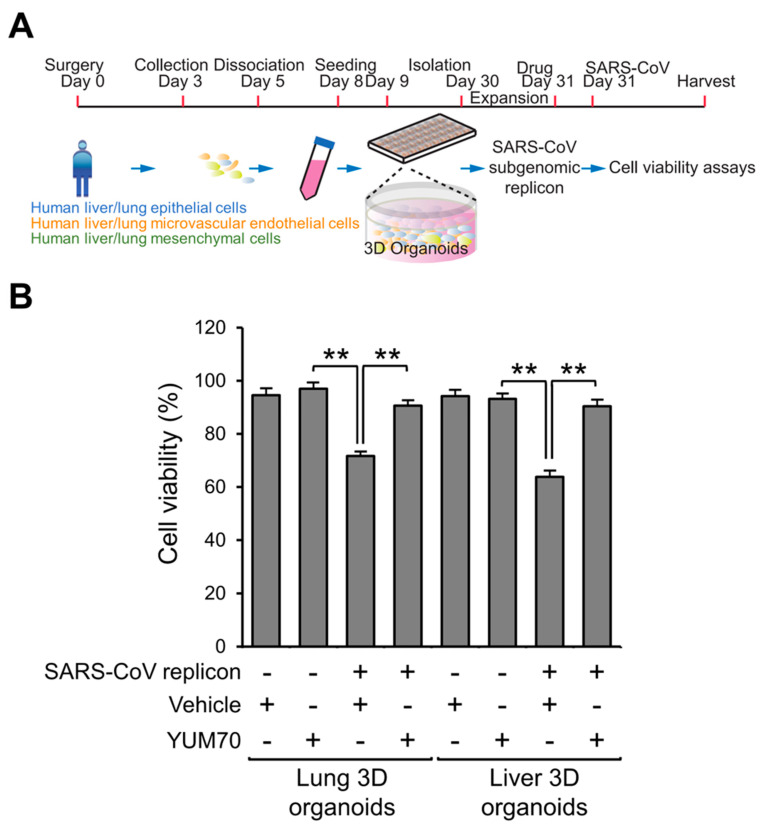
YUM70 recuses cell viability in multi-cellular human lung and liver 3D organoids when transfected with the SARS-CoV replicon. (**A**) Schematic diagram of the multi-cellular 3D organoid-based screenings assay. (**B**) Human lung and liver 3D organoids were transfected with subgenomic SARS-CoV replicon for 24 h, followed by treatment of YUM70 at 5 μM for 24 h. Cell viability was measured by XTT assay and shown in the graph (*n* = 6). Data are presented as means ± S.D. ** *p* ≤ 0.01 (Student’s *t*-test).

To evaluate the effect of YUM70 in a more physiologically relevant model system, we utilized a complex 3D organoid model composed of human lung and liver cells to examine the impact of YUM70 treatment in the context of SARS-CoV-2 infection. The multi-cellular human lung and liver 3D organoids were generated by combining various human cells, such as lung or liver epithelial, microvascular endothelial, and mesenchymal cells, in a specialized growth medium and environment. Subsequently, a sub-genomic replicon of SARS-CoV was expressed in the organoids to simulate SARS-CoV infection (Figure 4A). This resulted in a significant reduction in cell viability, as determined by the XTT assay, in both the lung and liver organoid model systems (Figure 4B). However, treatment with YUM70 rescued the decline in cell viability caused by the SARS-CoV sub-genomic replicon in both the lung and liver organoids (Figure 4B). Importantly, treatment with YUM70 alone did not affect cell viability in either the lung or liver organoids, indicating that the drug was not toxic and did not induce an increase in cell viability on its own (Figure 4B). These findings demonstrate that YUM70 could block the cytopathic effects of the SARS-CoV sub-genomic replicon in a physiologically relevant model system of human lung and liver 3D organoids.

### 3.5. YUM70 Treated Mice Show Trends of Ameliorated Weight Loss, Morbidity and Lung Damage Following SARS-CoV-2 Infection

**Figure 5:**
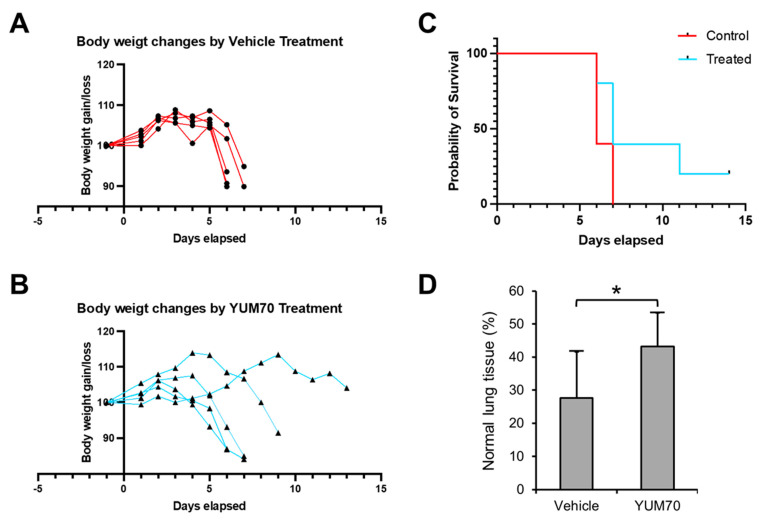
YUM70 ameliorates weight loss, mortality and lung damage following SARS-CoV-2 infection in mice. K18-hACE2 transgenic mice were infected with SARS-CoV-2 virus and treated with vehicle control (**A**) or YUM70 (**B**) daily (one day before infection and five times a week post-infection). Body weight was monitored daily and is shown in the graphs (*n* = 5). (**C**) Kaplan–Meier survival curves of mice infected with SARS-CoV-2 and treated with vehicle control or YUM70. (**D**) Percentage of normal lung tissues from mice infected with the SARS-CoV-2 and treated with vehicle control or YUM70 for five days. Data are presented as means ± S.D. * *p* ≤ 0.05 (Student’s *t*-test).

**Figure 6:**
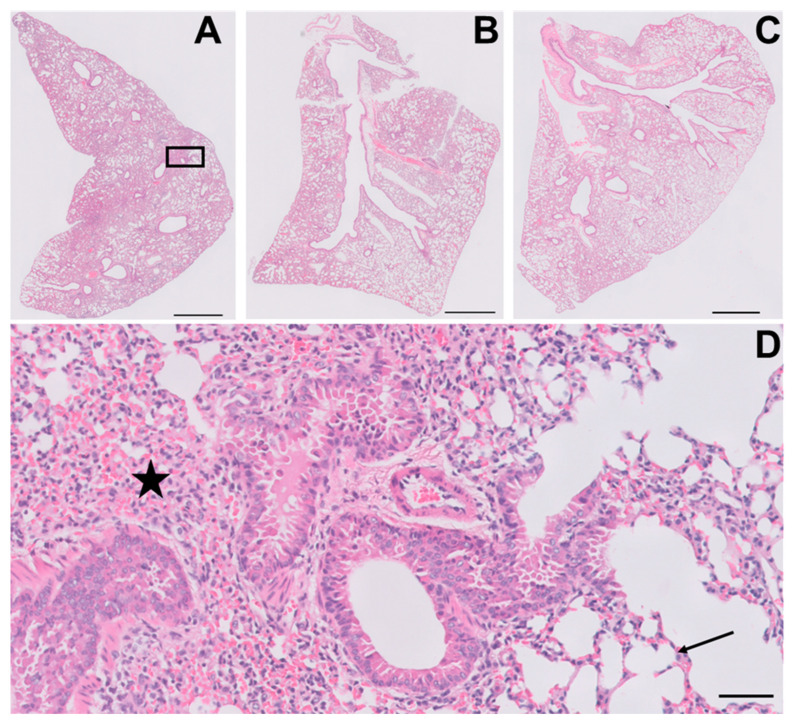
YUM70 reduces lung pathology in mice infected with the SARS-CoV-2. K18-hACE2 transgenic mice were infected with SARS-CoV-2 virus and treated with vehicle control or YUM70 daily. On day 3, the mice’s lungs were fixed in buffered formalin, embedded in paraffin, and 4-micron thick histological sections stained with hematoxylin and eosin were scanned and digitized. A representative whole-mount view of lungs from vehicle-treated mice is shown in (**A**), and YUM70-treated mice are shown in (**B**,**C**). (**D**) is a higher magnification of the area under the rectangle in A. The star shows an area with inflammation associated with alveolar thickening and collapse. The arrow points to a normal alveolar septum. Scale bars in A, B, and C represent 100 microns. The scale bar in D represents 50 microns.

Next, we tested the effect of YUM70 on K18-hACE2 transgenic mice infected with the SARS-CoV-2 virus. The pharmacokinetics of YUM70 were characterized in our previous study, and the injection dosage of 30 mg/kg was determined to be safe and effective in the xenograft mouse model [25]. The K18-hACE transgenic mice were administered either YUM70 (30 mg/kg) or vehicle control daily, and their body weight was monitored each day. We also assessed the survival of the mice and plotted the results using a Kaplan–Meier survival curve. YUM70 treatment led to reduced weight loss and a trend towards longer survival in infected mice compared to the control mice (Figure 5A–C). To further investigate the effects of YUM70 on the lungs, a critical organ involved in SARS-CoV-2 infection, including the lung tissues of infected mice treated with DMSO (controls) or YUM70 were fixed in buffered formalin, embedded in paraffin, and 4 micron-thick histological sections were stained with hematoxylin and eosin for histopathological examination. As expected, the lungs of the control mice infected with the virus showed significant damage and inflammation (Figure 6A,D), which was reduced by treatment with YUM70 (Figure 6B,C). The percentage of the total lung area examined that was histologically normal and statistically higher in mice treated with YUM70 than in untreated controls (Figure 5D), which showed extensive inflammation and alveolar collapse (Figure 6D). Taken together, these results suggest that YUM70 may effectively block SARS-CoV-2 infection in mice and reduce deleterious effects in critical organs such as the lung, which is consistent with reduced weight loss and improved survival.

## 4. Discussion

Since the recent discovery that the chaperone protein GRP78 is a host auxiliary factor for SARS-CoV-2 infection [15], evidence has been accumulating that GRP78 is not only central for the infectious cycle of diverse viruses, from entry and replication to assembly and exit, but is also critical for SARS-CoV-2 infection [4,24]. Importantly, serum GRP78 levels were found to be significantly higher in COVID-19 patients compared to the control groups, and leukocyte surface expression of GRP78 was increased in severe COVID-19 cases [20,30]. Recent studies further implied that GRP78 could be a good therapeutic target for patients infected with SARS-CoV-2 who are of older age, obese and diabetic [31]. With the rapid emergence of SARS-CoV-2 variants that have mutations in the spike protein that present challenges to the current vaccine strategy, there is an urgent need to identify cellular targets and therapeutics that can bypass these obstacles. Interestingly, recent computational analyses revealed that GRP78 could bind to the SARS-CoV-2 spike from different variants, including alpha, beta, delta, delta+, lambda, C36, and Omicron [32,33], suggesting that, in targeting GRP78, a critical host cell co-receptor may be effective in fighting the pandemic. Here, consistent with the computer modeling predictions, we demonstrated the effectiveness of the GRP78 inhibitor, YUM70, in blocking the entry of pseudo-viral particles bearing different variants of the SARS-CoV-2 spike protein. This suggests that the inhibitor’s effectiveness is not limited to a particular variant and could potentially suppress the spread of emerging SARS-CoV-2 variants.

Importantly, in this study, we found that YUM70 effectively reduced live SARS-CoV-2 virus infection in vitro without affecting cell viability, as confirmed by a plaque reduction assay in Vero E6 ACE2 cells. Moreover, our findings also shed light on the mechanism underlying YUM70’s ability to inhibit SARS-CoV-2 infection. We found that YUM70 could reduce both exogenous and endogenous spike protein production, indicating that GRP78 is not only important for the binding of spike protein to the cell surface but also essential for its production during active infection. We also observed a decrease in the endogenous N protein level in infected cells treated with YUM70, which is likely due to general translational arrest.

One of the significant findings of this study was that YUM70 could rescue the viability of multicellular human-derived lung and liver 3D organoids afflicted by the expression of SARS-CoV-2 replicon. The use of organoids is important because they can recapitulate more physiologically relevant conditions of the human lung and liver. This study’s findings suggest that YUM70 could be effective against SARS-CoV-2 infection in different cell types and have broad-spectrum antiviral activity against SARS-CoV-2 in different organs and tissues. In addition to the in vitro and 3D organoid models, we also utilized a transgenic mouse model to study the effect of YUM70 on SARS-CoV-2 in vivo. We observed that YUM70 treatment could effectively alleviate weight loss and prolong the lifespan of mice infected with a live SARS-CoV-2 virus compared to control-treated mice. However, further studies with larger sample sizes are needed to confirm these promising results. These observations are in agreement with our finding that YUM70 treatment can significantly reduce the lung damage caused by the infection of SARS-CoV-2 in the transgenic mouse model. Overall, our study suggests that the inhibition of GRP78, in general, may be a promising approach to combat SARS-CoV-2 infection. A wide range of GRP78 inhibitors have been reported, including natural products, synthetic molecules, specific peptides, and monoclonal antibodies [4,7,21,25,34,35,36,37]. Our study supports the further investigation of GRP78 inhibition as a viable approach with which to treat COVID-19 and the development of GRP78 inhibitors for the treatment of viral infections and other diseases that depend on GRP78.

## Data Availability

The data that support the findings of this study are available from the corresponding author upon reasonable request.
